# Overexpression of Cathepsin Z Contributes to Tumor Metastasis by Inducing Epithelial-Mesenchymal Transition in Hepatocellular Carcinoma

**DOI:** 10.1371/journal.pone.0024967

**Published:** 2011-09-22

**Authors:** Jian Wang, Leilei Chen, Yan Li, Xin-Yuan Guan

**Affiliations:** 1 Department of Clinical Oncology, The University of Hong Kong, Hong Kong, China; 2 State Key Laboratory of Oncology in Southern China, Cancer Center, Sun Yat-sen University, Guangzhou, China; University of Birmingham, United Kingdom

## Abstract

The aim of this study was to characterize the oncogenic function and mechanism of Cathepsin Z (*CTSZ*) at 20q13.3, a frequently amplified region in hepatocellular carcinoma (HCC). Real-time PCR were used to compare *CTSZ* expression between paired HCC tumor and non-tumor specimens. *CTSZ* gene was stably transfected into HCC line QGY-7703 cells and its role in tumorigenicity and cell motility was characterized by soft agar, wound-healing, transwell invasion and cell adhesion assay, and tumor xenograft mouse model. Western blot analysis was used to study expression of proteins associated with epithelial-mesenchymal transition (EMT).

Upregulation of CTSZ was detected in 59/137 (43%) of primary HCCs, which was significantly associated with advanced clinical stage (*P* = 0.000). Functional study found that CTSZ could increase colony formation in soft agar and promote cell motility. Further study found that the metastatic effect of *CTSZ* was associated with its role in inducing epithelial-mesenchymal transition (EMT) by upregulating mesenchymal markers (fibronectin and vimentin) and downregulating epithelial markers (E-cadherin and α-catenin). In addition, CTSZ could also upregulate proteins associated with extracellular matrix remodeling such as MMP2, MMP3 and MMP9. Taken together, our data suggested that *CTSZ* was a candidate oncogene within the 20q13 amplicon and it played an important role in HCC metastasis.

## Introduction

Hepatocellular carcinoma (HCC) is one of the most malignant cancers especially in Asian countries, and its poor prognosis is mainly due to metastasis after excision [1]. Genomic aberration, which leads to altered expression of genes within the aberration region, contributes greatly to the hepatocarcinogenesis. Using comparative genomic hybridization (CGH), chromosomal alterations including gain of 1q, 6q, 8q, 17q and 20q, and loss of 4q, 8p, 13q, 16q and 17p have been frequently detected in HCC [2]–[4]. It is believed that frequently amplified region may contain one or more oncogenes that play important roles in cancer development and progression. Identification and characterization of oncogene is one of the key issues in understanding the mechanism of tumor development and developing therapies against cancers [5]. Oncogenes often encode proteins that participate in signal transduction and intracellular signaling and can be activated mainly by overexpression, which can be accomplished in a number of ways including gene amplification.

In this study, a cathepsin family member cathepsin Z (*CTSZ*), located in a frequently amplified region at 20q13.3, which is related to the enhancement of metastasis in various cancers [6], was identified to be upregulated in 43% of HCC samples. *CTSZ* was also reported to be upregulated in gastric cancer and play a role in tumor development [7]. However the role of *CTSZ* in HCC metastasis has not been studied till now. In the present study, we found that *CTSZ* had a strong oncogenic ability and was able to increase cell motility and promote metastasis through the downregulation of epithelial markers E-cadherin and α-catenin, and upregulation of mesenchymal markers fibronectin and vimentin. In addition, *CTSZ* could upregulate expressions of MMP2, MMP3 and MMP9, which have been reported as metastasis contributors through their protease function targeting the extracellular matrix [8].

## Results

### CTSZ is frequently upregulated in HCC and the upregulation is correlated with poor survival of HCC patients

**Figure 1:**
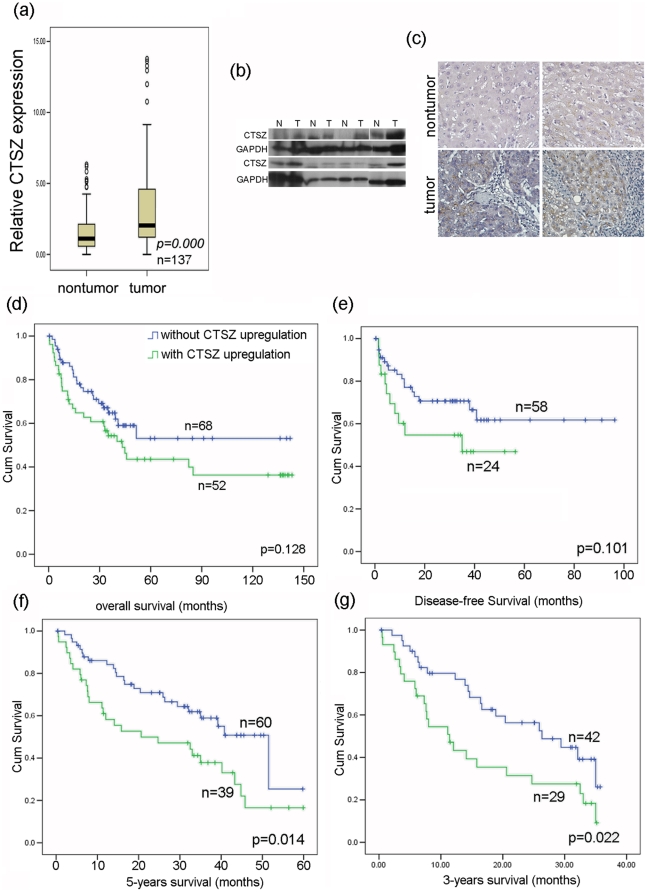
CTSZ is frequently upregulated in HCC. (***a***) The expression level of CTSZ relative to 18S rRNA was compared between nontumorous and tumor tissues in 137 HCCs using quantitative PCR. Expression of CTSZ in tumor tissues was significantly higher than that in nontumorous tissues (*P* = 0.000, n = 137). (b) Representative pictures of CTSZ protein expression in randomly selected paired HCC nontumor and tumor tissues, GAPDH was used as endogenous control. CTSZ was upregulated in most of the HCC tumor tissues compared to paired nontumor tissues. (c) Representative pictures of immunohistochemistry results of CTSZ subcellular localization. N: nontumor, T: tumor. Upregulation of CTSZ in tumor tissues was again found and the staining was only observed in liver cells rather than the surrounding lymphocytes. Overall Survival (***d***), disease free survival (***e***), 5-year survival (***f***) and 3-year survival (***g***) of HCC patients with or without CTSZ upregulation were compared using Kaplan-Meier method. Results indicated that the upregulation of CTSZ was significantly correlated to worse prognosis and shortened survival (*P* = 0.024).

CTSZ mRNA expression level was examined in 137 pairs of human HCC and their corresponding nontumorous liver tissues by qPCR. The result found that the upregulation of CTSZ was detected in 59/137 (43.7%) of the HCC cases, compared with their paired nontumorous liver tissues. The RQ of CTSZ in tumor and nontumor were then subjected to the following statistical analysis, and the result confirmed that the expression level of CTSZ was significantly higher in tumor tissues than that in their paired nontumorous liver tissues (*P* = 0.000, n = 137, Fig. 1*a*). The upregulation of CTSZ was also observed in the protein level by western blot assay, representative results were shown in Fig. 1b. Using immunohistochemistry method, we were able to determine the subcellular localization of CTSZ in HCC tissues. As shown in Fig. 1c, CTSZ staining was only found in liver cells, no staining can be found in the surrounding lymphocytes despite of their abundant existence. Using Kaplan-Meier method, overall survival, disease free survival, 5-year survival and 3-year survival of patients with or without CTSZ upregulation were compared. The results showed that the upregulation of CTSZ was significantly correlated with poorer 5-year (*P* = 0.014, Fig. 1f) and 3-year (*P* = 0.022, Fig. 1g) survival of HCC patients. Although not significant, CTSZ overexpression was still correlated with shorter overall survival (Fig. 1d) and disease free survival (Fig. 1e) time of HCC patients, and the reason of not significant correlation with disease free survival maybe due to some disease free survival information was not available.

### Overexpression of CTSZ correlates with advanced tumor stage

The correlation between CTSZ upregulation and clinical-pathological features including gender, age, HBsAg, serum AFP, tumor size, cirrhosis, adjacent organs invasion, reccurrence or metastasis, tumor encapsulation and tumor stage were studied. The result showed that the upregulation of CTSZ was significantly associated with the serum AFP (*P* = 0.001, Table 1). CTSZ upregulation was also found to be significantly correlated with advanced tumor stage (P = 0.000, Table 1). Although we failed to found significant correlation between CTSZ upregulation and metastasis related features, we did found that higher percentage (51%) of patients without tumor encapsulation has got CTSZ upregulation than that (34.8%) of patients with encapsulation (Table 1).

**Table 1:** Clinico-pathological Correlation of CTSZ Expression in HCC.

		CTSZ expression		
Clinicopathological features	Number (n)	Not upregulated	upregulated	P*
**Gender**				
Male	115	64 (55.7%)	51 (44.3%)	
Female	22	14 (63.6%)	8 (36.4%)	0.326
**Age**				
<60	108	60 (55.6%)	48 (44.4%)	
> = 60	29	18 (62.1%)	11 (37.9%)	0.340
**HbsAg** †				
Negative	30	20 (66.7%)	10 (33.3%)	
Positive	89	46 (51.7%)	43 (48.3%)	0.112
**Serum AFP** (ng/ml)§				
<500	69	30 (43.5%)	39 (56.5%)	
> = 500	49	36 (73.5%)	13 (26.5%)	***0.001***
**Tumor size** (cm)‡ ^,^ §				
<5	36	21 (58.3%)	15 (41.7%)	
> = 5	85	46 (54.1%)	39 (45.9%)	0.694
**Cirrhosis** §				
Absent	39	22 (56.4%)	17 (43.6%)	
Mild	35	18 (51.4%)	17 (48.6%)	
Moderate	31	19 (61.3%)	12 (38.7%)	
Severe	11	8 (72.7%)	3 (27.3%)	0.621
**Adjacent Organs Invasion** §				
Negative	81	49 (48.2%)	32 (51.8%)	
Positive	43	27 (38.9%)	16 (61.1%)	0.848
**Reccurrence or metastasis** §				
Negative	79	47 (60.5%)	32 (39.5%)	
Positive	45	29 (64.4%)	16 (35.6%)	0.702
**Tumor encapsulation** §				
Absent	51	25 (49.0%)	26 (51.0%)	
Present	66	43 (65.2%)	23 (34.8%)	0.091
**Tumor stage (TNM)** §				
Stage I	55	38 (69.1%)	17 (30.9%)	
Stage II	29	7 (24.1%)	22 (75.9%)	
Stage III	35	21 (60.0%)	14 (40.0%)	***0.000***

*: Two-sided χ^2^ test.

† : Hepatitis B surface antigen;

‡ : Tumor size was measured by the length of the largest tumor nodule;

§ : Partial data is not available, and statistic was based on available data;

### Overexpression of CTSZ increases the colony formation in soft agar

**Figure 2:**
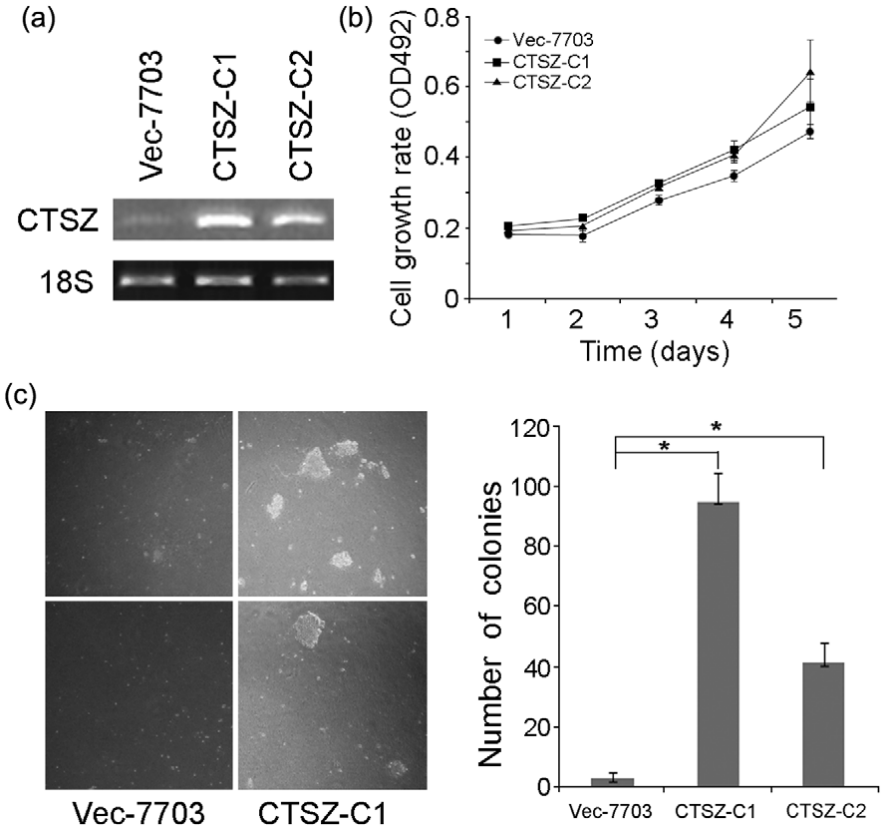
CTSZ processes strong tumorigenicity. (***a***) Expression of CTSZ in stably transfected QGY-7703 clones (C1 and C2) was detected by RT-PCR. Empty vector-transfected cells (Vec) were used as control. (***b***) XTT assay showed that no obvious difference was observed in cell growth rate between *CTSZ*-transfectants and Vec-7703 cells. (***c***) Representatives of colony formation in soft agar (left). The frequency of colony formation between *CTSZ*-C1, CTSZ-C2 and Vec-7703 cells was summarized in the right panel. Data were collected from three independent experiments. * *P* = 0.000.

To investigate the oncogenic ability of *CTSZ*, *CTSZ* was stably transfected into QGY-7703 cells and its expression was confirmed by RT-PCR (Fig. 2*a*). Cell growth assay showed that the cell growth rate was similar between *CTSZ*-7703 and Vec-7703 cells (Fig. 2*b*). Soft agar assay showed that the frequencies of colony formation in *CTSZ*-transfectants were significantly higher than that in Vec-7703 cells (*P*<0.05, Fig. 2*c*).

### CTSZ enhances cell migration and invasion while reduces cell adhesion

**Figure 3:**
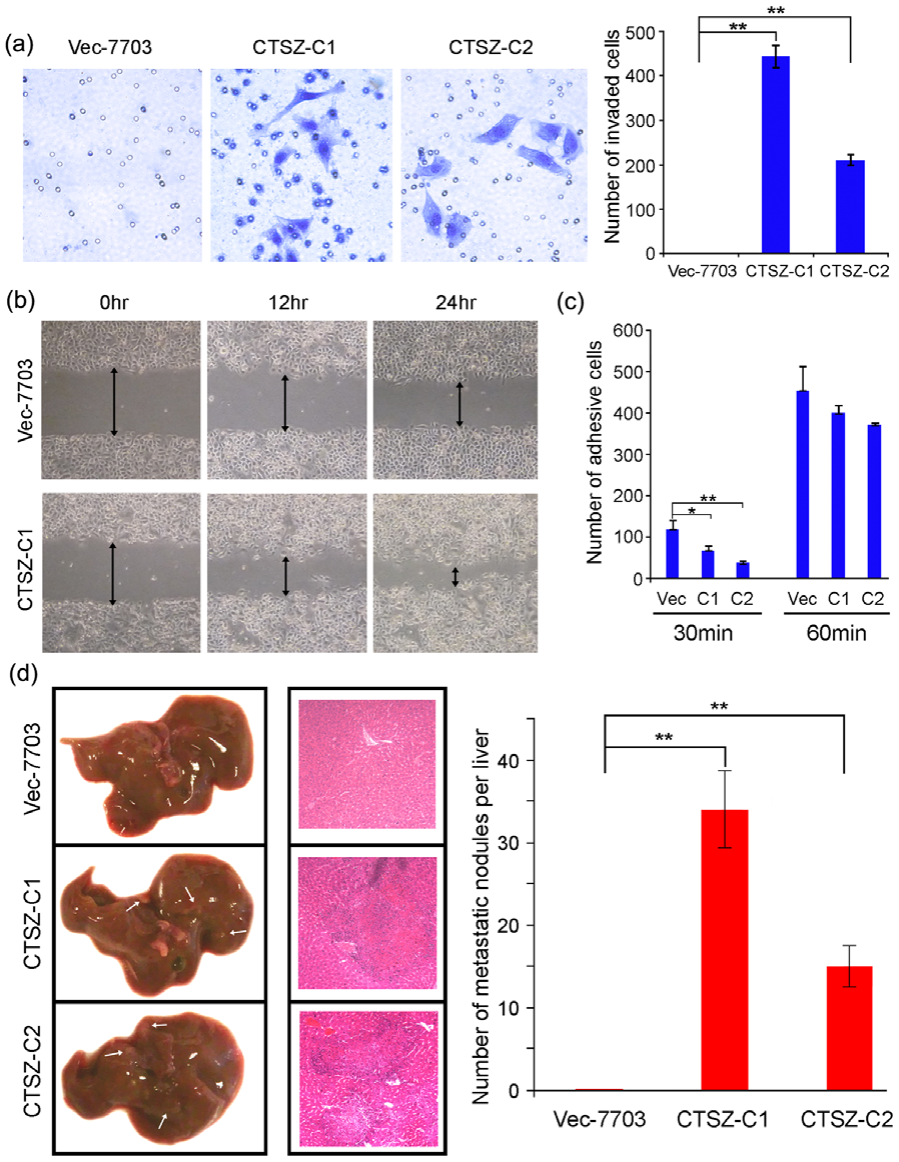
CTSZ promotes cell motility in HCC cells. (***a***) Representative images of the Matrigel invasion assay. The cells that invaded through the Matrigel were fixed and stained with crystal violet (200×magnification). Number of invaded cells was counted in 10 fields under 20× objective lens and summarized in the right panel. **P = 0.000. (***b***) The cell migration rate between CTSZ-C1 and Vec-7703 cells was compared by wound-healing assay. Microscopic observation was recorded at 0, 12 and 24 hours after scratching the cell layer. (***c***) Cell adhesion was assessed by seeding 1000 cells into 96-well plate, and washed with PBS at 30 and 60 minutes. Cells that adhesive to the plate were then stained with crystal violet and counted. Data were collected from three independent experiments. *P = 0.021, **P = 0.002. (***d***) Representatives of metastatic nodules on the surface of the liver in SCID mice induced by injecting 3×10^5^ cells (either *CTSZ*-7703 or Vec-7703 cells) intravenously. Mice were sacrificed one month after injection, and H&E staining of liver were shown in the middle panel. Nodules formed in livers were counted and summarized in the right panel. **P = 0.000. Data were collected from three independent experiments.

To investigate the effect of *CTSZ* overexpression on cell invasion, transwell invasion assay was performed using chamber coated with a thin layer of extracellular matrix, and the results showed that overexpression of *CTSZ* substantially enhanced the invasiveness of HCC cells, as indicated by a marked increase in the number of invaded cells (Fig. 3*a*). In addition, we found that *CTSZ*-7703 cells displayed a significant increase in cell migration ability compared with Vec-7703 cells by wound healing assay (Fig. 3*b*). Cell adhesion assay also found that both *CTSZ*-transfectants exhibited significant decreased adhesive ability compared to the vector-transfected cells at 30 mins timepoint (Fig. 3*c*), although the difference at 1 hour time point was no longer significant. This phenomenon was reasonable for that all the cells would attach at last.

### CTSZ promotes tumor metastasis in SCID mice

To further study the in vivo effect of *CTSZ* overexpression on tumor metastasis, an experimental metastasis assay was used to compare the metastatic nodules formed in lungs and livers of SCID mice following inoculation with *CTSZ*-7703 cells or Vec-7703 cells by tail vain injection. One month after injection, mice were sacrificed and lungs and livers were captured. No metastatic nodules were found in lungs in all tested animals. However, metastatic nodules on the surface of the liver was only observed in *CTSZ*-7703 cells, suggesting that *CTSZ* conferred the cells higher mobility which enabled them to extravasate from circulation and form metastatic nodules in liver (Fig. 3*d*). Histological studies confirmed that the lesions were caused by the extravasation, and the subsequent tumor growth of *CTSZ*-transfected HCC cells into the livers (Fig. 3*d*).

### CTSZ promotes metastasis by inducing EMT

**Figure 4:**
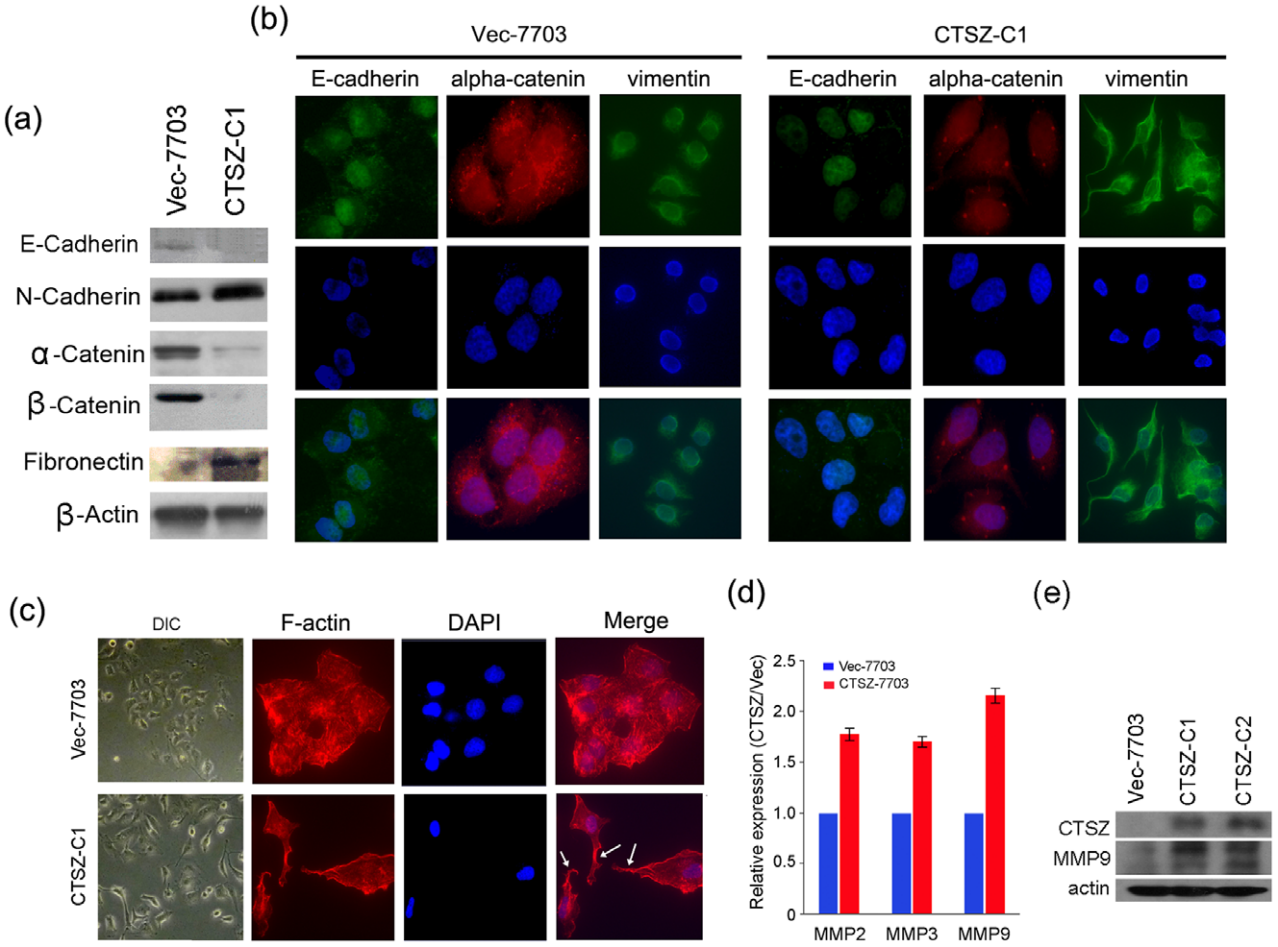
Overexpression of CTSZ induces epithelial-mesenchymal transition and upregulates MMP2, MMP3 and MMP9. (***a***) Expressions of epithelial markers (E-cadherin, α-catenin and β-catenin) and mesenchymal markers (N-cadherin and fibronectin) were compared by Western blot analysis between *CTSZ*-7703 and Vec-7703 cells. Beta-actin was used as loading control. (***b***) Downregulation of epithelial markers (E-Cadherin and α-catenin) and upregulation of mesenchymal marker vimentin were detected by immunofluorescence in *CTSZ*-7703 cells compared to Vec-7703 cells. (***c***) Representatives of cell morphological changes after introduction of CTSZ into QGY-7703 cells (left). F-actin was stained with fluorescein isothiocyanate conjugated phalloidin (right) and the formation of filopodia (indicated by arrows) could be detected in CTSZ-7703 cells but not in Vec-7703 cells. (***d***) Overexpression of CTSZ could upregulate MMP2, MMP3 and MMP9 expression detected by qPCR. (e) CTSZ overexpression and MMP9 upregulation in CTSZ-C1 and CTSZ-C2 was confirmed by western blot. **P = *0.000*. All data were collected from three independent experiments.

To explore the mechanism through which CTSZ conferred higher mobility on QGY-7703 cells, expression of epithelial markers (E-cadherin, α-catenin and β-catenin) and mesenchymal markers (Fibronectin, N-cadherin) were analyzed by Western blotting and immunofluorescence assays (IF). Western blot analysis showed that E-cadherin and α-catenin, and β-catenin were downregulated, while Fibronectin and N-cadherin was upregulated in *CTSZ*-7703 cells compared with Vec-7703 cells (Fig. 4*a*). Consistent with Western blotting data, downregulation of E-cadherin and α-catenin, and upregulation of Vimentin were also detected in *CTSZ*-7703 cells by IF (Fig. 4*b*). Interestingly, the morphology of *CTSZ*-7703 cells was changed from circular-like epithelial cells to elongated spindle-like mesenchymal cells, which also contributed to the elevated cell mobility (Fig. 4*c*). In addition, isothiocyanate conjugated phalloidin staining found that *CTSZ*-7703 cells formed philopodia.

### Overexpression of CTSZ upregulates MMP2, MMP3 and MMP9

As shown above, overexpression of CTSZ reduced cell adhesion, suggesting some of extracellular matrix remodeling proteins such as MMPs might be upregulated by CTSZ. Compared to Vec-7703 cells, upregulations of MMP2, MMP3 and MMP9 were detected in *CTSZ*-7703 cells by qPCR (Fig. 4*d*). MMP9 upregulation in CTSZ-C1 and CTSZ-C1 was further confirmed by western blot (Fig. 4*e*). MMP9 was shown as double bands here, and from previous study we know that the smaller band which is around 75KD represents the active form of MMP9. So the results here showed that not only MMP9 was upregulated in the CTSZ overexpressed cell line, but the active form of MMP9 also increased.

## Discussion

In this study, we found that CTSZ, within a frequently amplified region at 20q13.2, was frequently upregulated in HCC. Ectopic expression of CTSZ could increase cell motility, implying that CTSZ might play an important role in HCC invasion and metastasis. Metastasis is a multistep process which contains migration to adjacent tissue, intravasation to circulation, survival in circulation, extravasation to secondary tissues, and colony formation at the secondary tissues [9]. Factors affect one or more steps may finally enhance or inhibit the outcome of metastasis. The mechanism that initiates or promotes tumor metastasis has long been a key issue being studied for developing more effective therapies. Among those metastasis-related genes, cathepsin family members stand out by playing unique roles. CTSZ belongs to cathepsin family, which has been closely associated with cancer progression. Cathepsin B promotes progression and metastasis in mammary cancer, and the enhanced expression of cathepsin inhibitor Stefin A inhibits distant metastasis in primary breast cancer [10]. Cathepsin D is an independent marker of poor prognosis in breast cancer correlated with the incidence of clinical metastasis [11], and downregulation of Cathepsin D inhibits tumor growth and experimental metastasis of human breast cancer cells [12]. In osteosarcoma, Cathepsin K has been identified as an indicator of metastasis, and patients with lower expression of Cathepsin K have better prognosis [13].

Although with some limitations that this study was conducted by ectopic overexpression system, but not an inducible system, our results of CTSZ promoting cell metastasis remains consistent with other studies recently. Sevennich and colleagues proved that combined inhibition of Cathepsin B and Cathepsin Z inhibited breast cancer metastasis in mice [14]. Kraus et al found downregulation of Cathepsin X induced cellular senescence and inhibition of cell invasion as well [15]. A cDNA microarray study also illustrated that CTSZ was one of those upregulated genes related with melanoma malignancy [16]. In our study, introduction of CTSZ into HCC cell line QGY-7703 could induce the conversion of cobblestone-like epithelial morphology into spindle-shape mesenchymal morphology. These morphological and cell-cell contact changes might ultimately reflect on cell mobility and invasive ability. Consistent with the morphological change, some hallmark proteins of epithelial cells including E-cadherin, α-catenin and beta β-catenin have been reduced, meanwhile the mesenchymal cell markers fibronectin and N-cadherin have been upregulated. These results strongly suggested that the increasing motility conferred by CTSZ was via EMT. EMT was originally recognized as a critical step to metazoan embryogenesis and in defining structures during organ development. In the past decade, EMT has arisen to be recognized as an important process promoting tumor metastasis and malignancy. Expression of proteins that are characteristic of mesenchymal cells (e.g. vimentin, FSP1/S100A4, SNAI1, SNAI2, and stromelysin-3) and loss of epithelial markers (e.g. E-cadherin) correlates with tumor progression and poor prognosis [17]. Study shows that invasion of adenocarcinomas is accompanied by the release of single cells through EMT [18], suggesting that EMT plays a key role in malignant cell invasion. Several metastasis-related genes, such as HOXB7 [19], FOXC2 [20] and Twist [21] have been identified being able to initiate EMT these years. However, distinguished from the genes related with EMT mentioned above, Cathepsin Z existed as a secreted protein and no report showed it has transcriptional property, thus the EMT phenotype induced by CTSZ overexpression may not be due to the transcriptional regulation of EMT markers. Since CTSZ has been suggested to take part in proteinase degradation [22], the EMT related phenotype reported in our study could result from epithelial marker degradation, although further study was still needed to identify the exact substrates of CTSZ. A non-transcriptional EMT pathway has also been reviewed by Xu and colleagues [23].

Matrix metalloproteinases (MMPs) have long been associated with cancer-cell invasion and metastasis. MMPs are proteolytic enzymes and their basic mechanism of action is to degrade proteins in extracellular matrix. Activation of MMPs has been detected in almost all type of human cancer and closely correlated with advanced tumor stage, increased invasion and metastasis, and shortened survival time. MMP2, MMP3 and MMP13 have been found to promote invasion of cell lines through either collagen type I or optic nerve explants [24]. Inhibition of MMP9 expression by a ribozyme could reduce the number of metastatic nodules formed in the lungs of mice [25]. Therefore, another mechanism of *CTSZ* in tumor metastasis is associated with its role in reducing cell-cell adhesion by digesting the extracellular matrix ingredients, thus makes it easier for tumor cells to migrate to the adjacent tissues. The protease activity may also facilitate the intravasation and extravasation processes. The pro-metastatic role of *CTSZ* may serve as another clue to the development of cancer therapy, especially the inhibition of metastasis. As CTSZ is a secreted protein, it is highly possible to develop a neutralizing antibody for aborting its protease activity and thus slower or suppress the metastasis process.

## Materials and Methods

### Ethics statement

Human tissue samples used in this study were approved by the Committees for Ethical Review of Research involving Human Subjects at Cancer Center of Sun Yat-Sen University. Written informed consent were obtained from all participants involved in this study. Animal operation was carried out according to the protocols approved by the Committee on the Use of Live Animals in Teaching and Research (CULATR). License to conduct live animal experiments was approved by Hong Kong Department of Health (lisence no. (09-64) in DH/HA&P/8/2/3 Pt.10).

### Cell lines and tumor specimens

Human HCC cell line QGY-7703 was obtained from the Institute of Virology, Chinese Academy of Medical Sciences (Beijing, China). Forty-five HCC patients, who underwent hepatectomy for HCC at the Cancer Center of Sun Yat-Sen University (Guangzhou, China), were included in this study. None of these patients received preoperative chemotherapy or radiotherapy.

### RT-PCR and quantitative real-time PCR

Total RNA was extracted using TRIzol Reagent (Invitrogen, Carlsbad, CA), and reverse transcription was performed using an Advantage RT-for-PCR Kit (Clontech Laboratories, Mountain View, CA, USA) according to the manufacturer's instructions. For quantitative real-time PCR (qPCR) analysis, aliquots of double-stranded cDNA were amplified using a SYBR Green PCR Kit (Applied Biosystems, Foster City, CA) and an ABI PRISM 7900 Sequence Detector. The cycling parameters were 95°C for 30 s, 55°C for 1 min and 72°C for 2 min for 45 cycles, followed by a melting curve analysis. The threshold cycle (CT) was measured during the exponential amplification phase, and the amplification plots were analyzed by SDS 1.9.1 software (Applied Biosystems, Foster City, CA). Primers used are as followed, CTSZ: F-5′ AGAGTGCCACGCCATCC 3′, R-5′ CGCCCTTCCCATCCTTAT 3′; 18S: F-5′ CTCTTAGCTGAGTGTCCCGC 3′, R-5′ CTGATCGTCTTCGAACCTCC 3′; MMP2: F-5′ CTTCCAAGTCTGGAGCGATGT 3′, R-5′ TACCGTCAAAGGGGTATCCAT 3′; MMP3: F-5′ CAGTTTGCTCAGCCTATC 3′, R-5′ CAGAGTGTCGGAGTCCAG 3′; MMP9:F-5′ GGGACGCAGACATCGTCATC 3′, R-5′ TCGTCATCGTCGAAATGGGC 3′.

### In vitro oncogenic assays

To test the oncogenic function of *CTSZ*, full-length *CTSZ* sequence was amplified by PCR with primers listed in material and methods. Purified PCR product was cloned into pCDNA3.1(+) expression vector (Invitrogen, Carlsbad, CA), and then stably transfected into QGY-7703 cells (*CTSZ*-7703) with Lipofectamine2000 (Invitrogen, Carlsbad, CA) according to manufacturer's instruction. Clones (C1 and C2) with G418 resistance were selected and expanded. Empty vector-transfected QGY-7703 cells (Vec-7703) were used as control. Cell growth rate and colony formation in soft agar were carried out as described previously [26].

### Cell migration and invasion assays

Cell invasion assay was performed with precoated cell invasion kit (Chemicon International, Temecula, CA). Cells that had invaded through the extracellular matrix layer to the lower surface of the membrane were fixed with methanol and stained with crystal violet. Images of 3 randomly selected fields of the fixed cells were captured and cells were counted. Experiments were repeated independently three times. For the wound healing assay, cells were cultured to 90% confluency when a wound was made by tips. Pictures from the same area of wound were taken at 0, 12 and 24 hours under microscope after scraping.

### Tumor xenograft mouse model

Five-week-old male severe combined immunodeficient (SCID-Beige) mice were used for tumor xenograft mouse model. CTSZ-7703 (C1 or C2) or Vec-7703 cells (3×10^5^) were injected to SCID mice though tail vain, respectively (3 mice for each group). Mice were sacrificed 4 weeks after injection. The presence of tumor nodules was macroscopically determined, and the number of tumor nodules formed on the liver surfaces was counted. The livers were excised and embedded in paraffin. Sections (5 µm) of liver were stained with hematoxyline and eosin (H&E) to visualize the tumor structure. All animal procedures were performed in full accordance with CULATR-approved protocol.

### Western blot analysis

Western blot analysis was performed as described previously (9) with antibodies for CTSZ, vimentin, fibronectin, E-cadherin and β-actin (Santa Cruz Biotechnology, Santa Cruz, CA), N-cadherin, β-catenin and α-catenin (Cell Signalling Technology, Germany).

### Immunofluorescent (IF) microscopy

For IF staining, cells were seeded onto coverslips, incubated overnight and then fixed with 4% paraformaldehyde. Fixed cells were incubated with 1∶2000 fluorescein isothiocyanate-conjugated phalloidin (Sigma, St. Louis, MO) or antibodies as indicated. Cells were then mounted with Mounting Medium containing DAPI (Vector Laboratories, Burlingame, CA). Images were captured by a Leica DMRA fluorescence microscope (Leica, Wetzlar, Germany).

### Immunohistological chemistry staining

Tissue slides were dewaxed according to standard protocol. Briefly, slides were dewaxed in 70°C incubator followed by abosolute xylene rinse for 10 mins. Rehydration of slides were carried out by serially rinsing slides in 100%, 95%, 80% and 50% (v/v) ethanol for 5 mins each. Antigen retrieval was done by boiling slides in antigen retrieval buffer for 15 mins and cooling naturally. Primary antibody was added to the tissues on the slides and incubated at 4°C overnight. Secondary antibody was added for 1 hour after rinsing by PBS. DAB was used for staining.

### Statistical analysis

The SPSS statistical package for Window version 13 (SPSS, Chicago, IL) was used for data analysis. RQ of CTSZ in tumor and nontumor samples were compared using paired T-test. The clinicopathological features of patients with or without CTSZ upregulation were compared using Pearson's chi-square test for categorical variables. The survival curves of patients with or without CTSZ upregulation was calculated by Kaplan-Meier method. A p value less than 0.05 was considered statistically significant.
